# Digital technologies for climate-related health education, behavior and risk reduction: a systematic scoping review

**DOI:** 10.1038/s41746-025-01907-5

**Published:** 2025-08-02

**Authors:** Nathan B. Morris, Megan Barnes, Autumn Rybarczyk, Georgia K. Chaseling

**Affiliations:** 1 William J. Hybl Sports Medicine and Performance Center, Department of Human Physiology and Nutrition, University of Colorado, Colorado Springs, CO USA; 2 Sydney Nursing School, Faculty of Medicine and Health, The University of Sydney, Sydney, NSW Australia; 3 Heat and Health Research Centre, School of Health Sciences, Faculty of Medicine and Health, The University of Sydney, Sydney, NSW Australia

**Keywords:** Climate change, Health care

## Abstract

Digital technologies improve health outcomes, access to education and health care. However, their application in the context of climate change is limited. This scoping review aimed to identify the use of digital technologies for climate-related health education, behavior change and health risk reduction in a rapidly changing climate. After screening 20,342 titles, 24 studies published between 2012 and 2025, with 18,749 participants (~54% female), were included in the analysis. Digital technology was used to improve health education (*n* = 11 studies) health outcomes (*n* = 9 studies) or health behavior (*n* = 6 studies). Common climate change topics focused on general climate change (*n* = 6), heatwaves (*n* = 4), and earthquakes (*n* = 3). Commonly used technologies were virtual reality (*n* = 9), smartphone applications (*n* = 7) and online platforms (*n* = 4). While this field is still nascent, there is a clear opportunity to utilize digital technology to reduce the negative health impacts of climate change, with an emphasis on interdisciplinary research and co-designed technologies.

## Introduction

Climate change has been declared the greatest threat to human health of the 21st century^1^. The unrelenting impacts of extreme weather events on both human health and healthcare systems are increasingly overwhelming^2^, largely due to anthropogenic climate change. The rising frequency and intensity of heatwaves have exposed 78% of the global population to at least 31 days of extreme heat annually^3^, which contributes to over 489,075 deaths globally each year^4^. In parallel, heatwaves have led to ~490 billion labor hours lost annually^5^, significantly amplifying income losses, especially in countries with low Human Development Index scores, where wage workers are most vulnerable to economic instability^5^. Simultaneously, an increase in floods across the globe are resulting in thousands of excess deaths^6^. While collectively severe droughts, floods, wildfires, and hurricanes are threatening food and water security and sanitation, leading to malnutrition, increased risk of chronic diseases and weakened immunity^5,7^. The indirect threats of climate change on human health are also escalating, worsening global health inequalities and undermining the core pillars of human health. Health systems are under mounting pressure, with 27% of cities surveyed reporting fears that climate-related impacts could overwhelm their capacity^5^. The health impacts of climate change have only recently been prioritized by major health organizations, as exemplified by the first annual *Lancet Countdown on Health and Climate Change* published in 2015^8^. Despite the anthropogenic nature of climate change, many of the detrimental effects on human health are preventable. However, there is a critical need to find solutions to effectively reduce the burden of climate change on human health.

Globally, digital innovations are transforming healthcare, education, communication and connectivity. The World Health Organization’s Global Strategy on Digital Health emphasizes the importance of strengthening our healthcare systems through safe and robust implementation of digital health technologies that aim to reduce health inequities and improve the standard of care^9^. Indeed, advancements in digital technologies have already led to improvements in chronic disease detection and patient outcomes^10–12^, general health follow up visits^10^, and health interventions and lifestyle change^13^. In the last 5 years, particularly since the beginning of the COVID-19 Pandemic, the need to extend the reach and use of digital technologies in healthcare, work, and education settings has been emphasized to ensure adequate access to people restricted from leaving their home and/or working and living remotely. The accelerated implementation and use of digital technologies demonstrates their potential to transform the future of healthcare.

The application of digital technology has shown favorable results to improve access to healthcare, empower patients to effectively manage chronic conditions^10–12^, and as a medium for education and behavior change^10,14,15^. However, literature demonstrating the application of digital technology to climate change appears to be limited. With the global population both growing and aging^16^, increased migration and reduced access to care that is driven by floods, droughts, and wildfires^17^, traditional healthcare services will face mounting pressures. These compounding challenges may make it difficult for health systems to deliver timely, high-quality care, highlighting the need for innovative approaches to maintain resilience and effectiveness.

This gap of inaccessibility can be bridged by digital technologies that are not restricted by geographic location. However, implementing digital health solutions in the context of climate change does not come without challenges. People who live remotely, who often lack awareness of the threat of climate change^18^, are disadvantaged by limited infrastructure to support digital connectivity, yet are the most affected by climate change^5^. Furthermore, ineffective co-design (a collaborative process involving end users in the design and implementation of technologies that affect them) can lead to unsuitable and counterproductive technologies, particularly amongst vulnerable populations such as the elderly, people who come from culturally and linguistically diverse backgrounds^5,18^. This systematic scoping review aimed to identify digital technologies currently used in the context of climate change to reduce health risks and improve health and/ behavior change and how they have been designed and implemented. The primary aim was to identify which technologies were being used, under which climate change contexts (e.g., heatwaves, floods, air pollution), for which purposes (i.e., for health education or health purposes), and amongst which populations. Additionally, we included semantic analyses of the retrieved abstracts, outcome variables, and authors to provide further insights into the state of the field.

## Results

### Search overview

**Fig. 1:**
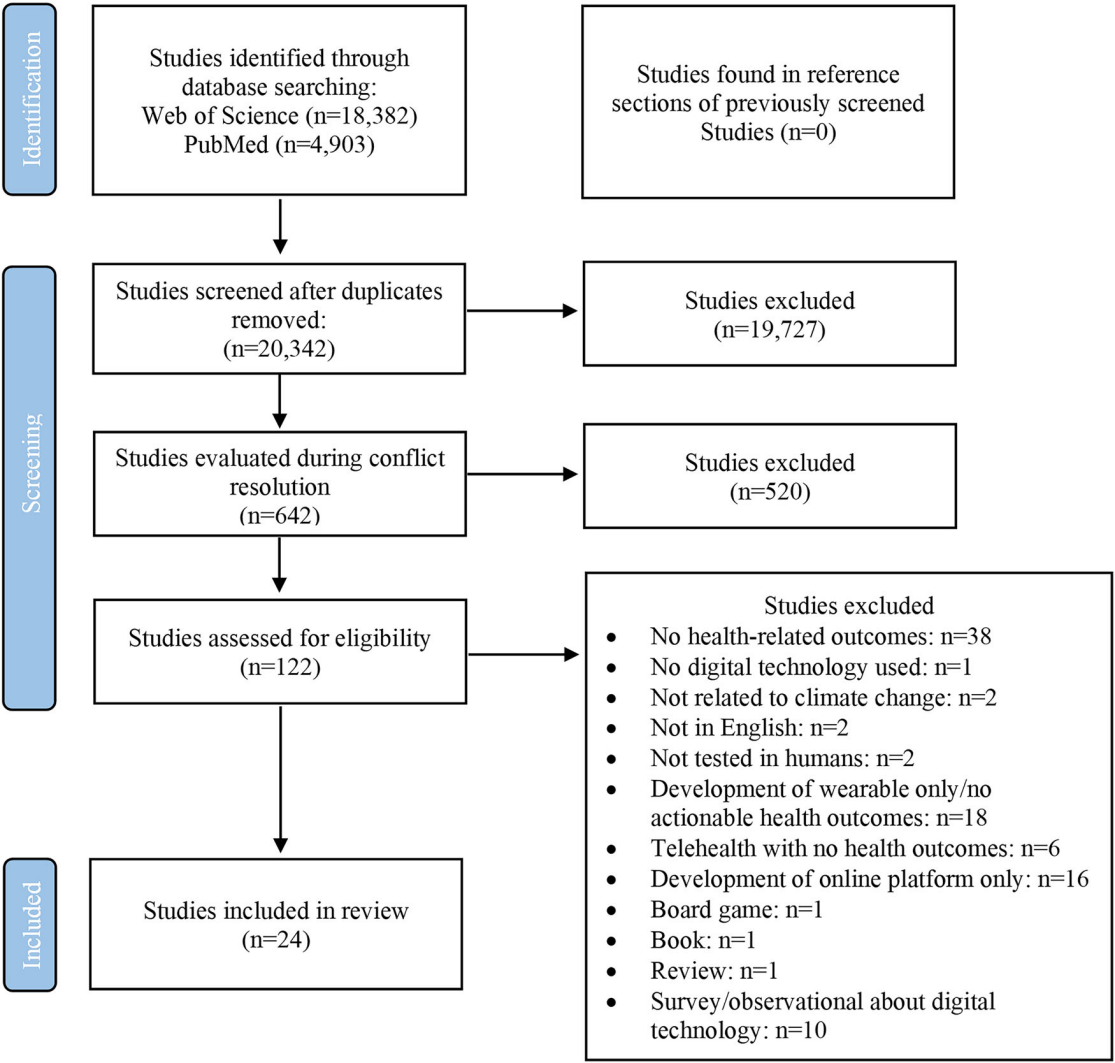
Flow diagram of the review screening process. Flow diagram of the review screening process showing the number (n) of studies originally identified, screened, assessed for eligibility with reason for exclusions.

The screening process for our search is detailed in Fig. 1. Following removal of duplicates between databases, 20,342 unique titles were screened. From these, 19,727 titles were further excluded. Following full text screening, 122 studies were assessed for eligibility, of which 96 were excluded because they had no health-related outcomes (*n* = 38), were not related to climate change (*n* = 2), there was no digital technology used (*n* = 1), not published in English (*n* = 2), not tested in humans (*n* = 2), focused on the development or design of a wearable only or did not provide actionable health outcomes (*n* = 18), used telehealth without the inclusion of health-related outcomes (*n* = 6), involved the development of an online platform only (*n* = 16), or were a type of paper excluded from our review (i.e., survey/book chapter) *n* = 13. This resulted in 24 studies included in the final analysis^19–42^.

### Study Characteristics

**Fig. 2:**
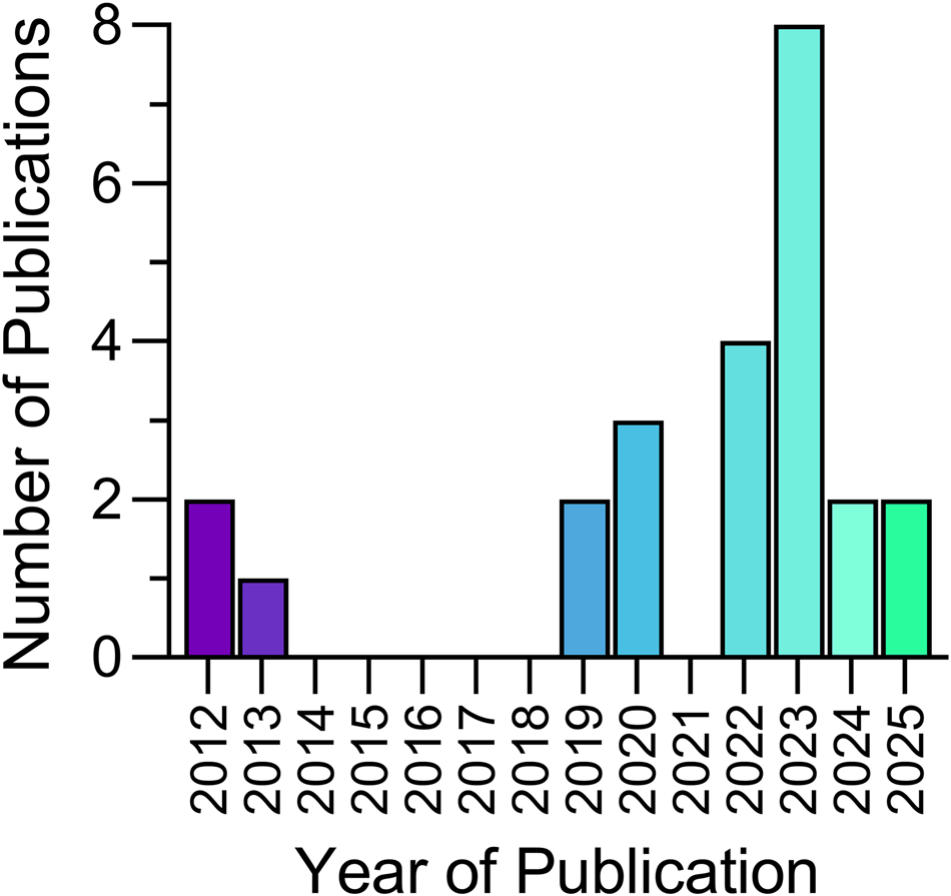
Study publication year. Number of articles and the year of publication for studies included in analysis.

**Fig. 3:**
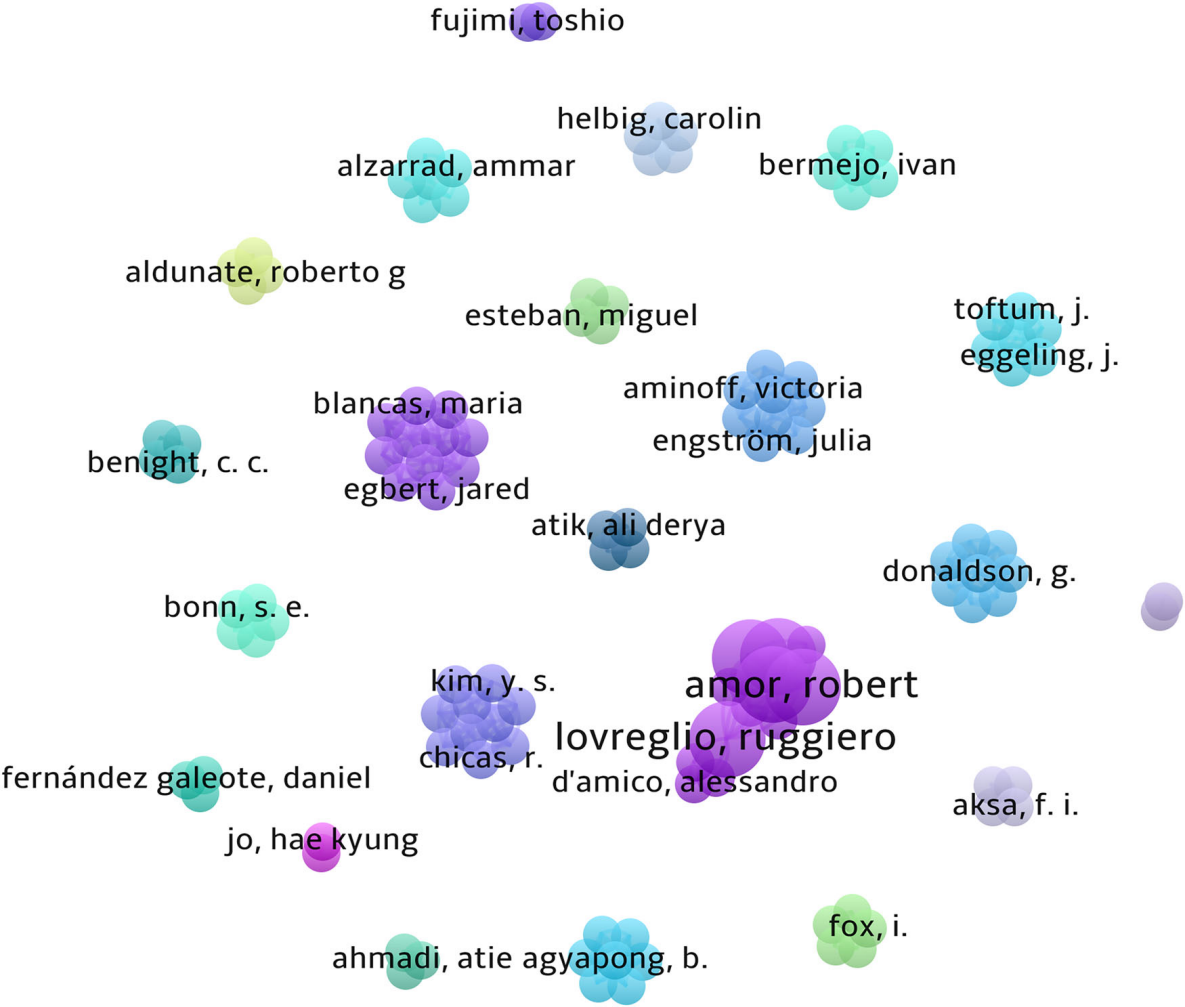
Author collaboration clusters of included papers. Each cluster represents the group of authors for each manuscript. Where author collaboration exists, the thickness of the line between clusters represents the strength of the collaboration. However, our analysis found no collaboration between any authors who have published from the included articles.

A summary of the study locations, year of publication, aim(s), design, and main findings are displayed in Supplementary Table 1. The first articles on record were published in 2012^36,39^. In the following 10 years only seven articles were published, until 2022–2025, where 15 articles were published collectively. When the search was performed on the 11th of March 2025, two articles had been published in 2025 (Fig. 2). Author collaboration clusters are shown in Fig. 3. In total, 123 people authored one document, with five authoring two documents. Publications originated from the United States (*n* = 5), Germany (*n* = 2), Italy (*n* = 2), Japan (*n* = 2), New Zealand (*n* = 2), Sweden (*n* = 2), Australia (*n* = 1), Canada (*n* = 1), Chile (*n* = 1), Finland (*n* = 1), Indonesia (*n* = 1), Iran (*n* = 1), Netherlands (*n* = 1), South Korea (*n* = 1) and Turkey (*n* = 1).

Table 1 shows the outcome measures and digital technology used within which climate change topics, categorized into either health outcomes, health education, behavior change or implementation. The areas of interest were using digital technology to improve health education (*n* = 11 studies), modifying health outcomes (*n* = 9 studies), followed by altering health behavior (*n* = 6 studies) and implementation (*n* = 7 studies). Three studies used co-design in the development of their technology^24,25,37^, 12 studies used their technology to prepare people for extreme weather events (i.e., flood or earthquake evacuations)^19–23,25–30^, and six studies used technology to help people manage their health during extreme weather events^25,33,35–37^. Digital technologies used (with some studies using more than one) included virtual reality (VR; *n* = 9), smartphone application (*n* = 7), gaming (*n* = 7), online platform (*n* = 4), wearable (*n* = 3), telehealth (*n* = 2) and text messaging (*n* = 1).

**Table 1:** Study Characteristics

Climate change Topic (*n*)	Digital Technology (*n*)	Primary Outcome
General climate change (6)	App (3)	- Health education - Health outcome - Behavior change - Implementation
	Telehealth (1)	- Health outcome
	Virtual reality/Gaming (1)	- Health education - Implementation
	Online platform/Gaming (1)	- Health education - Implementation
Heatwaves (4)	Wearable/App (2)	- Health outcome - Health education
	Telehealth (1)	- Health outcome - Implementation
	Virtual reality (1)	- Health education
Earthquakes (3)	Virtual reality/Gaming (3)	- Health education - Implementation
Flooding (3)	Virtual reality (2)	- Health education - Implementation
	Virtual reality/Gaming (1)	- Health education - Implementation
Air pollution (1)	Wearable (1)	- Health education - Behavior change
Wildfires (2)	Online platform/Gaming (1)	- Health education
	Test messaging (1)	- Health outcome
Hurricanes (2)	Virtual reality (1)	- Behavior change
	Online platform (1)	- Health outcome
Tsunami (1)	App (1)	- Behavior change
Pollen (1)	Online platform (1)	- Health outcome
Extreme cold (1)	App (1)	- Health outcome

Study characteristics of the climate change topic, the digital technology used, and the primary outcome variable categorized into either health education, health outcomes, behavior change or implementation.

### Study participants

Twenty-three of the 24 studies reported participant information. There was a total of 18,749 participants. Nineteen articles included information about participant sex, and from those, there were a total of 1533 participants: 703 (45.9%) were male, 825 (53.8%) were female, three were non-binary (0.2%) and two (0.1%) preferred not to specify their sex. The age of participants reported from 12 studies was 36 (7–87 years). Participant demographics ranged from primary and secondary school and university students to farm workers, people with diabetes and chronic obstructive pulmonary disease (COPD) and general population residing in the area where the study was undertaken (Supplementary Table 1).

### Study design

Seven studies employed various smartphone app-based interventions targeting health education/preparedness for and management of health outcomes (e.g., heat-related illness) during climate events. In some studies, the efficacy of the app was compared to ‘standard care’^34,38^ or control conditions without the use of an app^25,30^. Four studies used pre and post-questionnaires to assess the effectiveness of an intervention on health education^31^, or modify behavior or decision making^34,38^ employing either a within- or between-subject design. Other studies used apps to improve decision making, such as improving evacuation times during a tsunami by assessing the time to evacuation^30^ or monitoring biometrics and/or predicted thermal stress (two in the heat^25,35^ and one in the cold^27^) and providing actionable advice to remain in a healthy thermal state.

Nine studies used VR to understand the influence of immersive experiences to improve health education, climate change health-related awareness and modify behavior. Virtual reality was primarily used to prepare individuals for making decisions during climate events, with seven of the nine studies serving this purpose. In several studies, the effectiveness of exposure to VR or 3D simulations was compared to traditional information delivery methods, such as PowerPoint, or control conditions without VR^22,23,29,42^. Study designs mostly used pre- and post-test questionnaires to measure knowledge, attitudes, self-efficacy, and intentions. In most instances, the duration of VR engagement was not reported, however, in those studies that did, durations varied from 60 s to ~30 min. Several studies utilized immersive narrative experiences or interactive tasks where participants’ actions within the VR environment affected the outcomes they observed, particularly during VR that simulated evacuations. The study designs commonly incorporated elements of inquiry-based learning, multimedia, and gamification to engage participants and assess both cognitive and emotional impacts.

Digital technologies often employ gaming to improve health education and behavior using pre and post-test questionnaires, with most users engaging in the game once. The design of games varied, and was used through either VR or online platforms, including some specifically purposed for educational settings, which improved players’ knowledge about climate change and their decision-making abilities. Other games emphasized feedback to reinforce learning^20^, or incorporated storytelling elements^41^ to increase both knowledge and engagement. Game durations varied widely, ranging from a single 25-minute session to several weeks of gameplay. However, the length of time spent playing did not appear to significantly impact outcomes, with enjoyment and positive emotions toward climate change remaining consistent across different gameplay durations.

Three studies used wearables to measure human physiological and environmental parameters to improve health outcomes and education and modify behavior^25,32^. All studies employed a feedback system where people would become aware of their health status or surrounding environment based on the wearable and behaviors were monitored accordingly.

Two studies utilized telehealth interventions: one delivered eight weeks of cognitive behavioral therapy to alleviate climate-related anxiety^40^, while another provided nine months of remote monitoring for patients with COPD^33^, aiming to reduce exacerbations during hot weather. The telehealth platforms facilitated regular check-ins, remote consultations, and personalized feedback. Both studies demonstrated that telehealth interventions were significantly more effective than control groups, improving patient outcomes by providing continuous care, timely interventions, and tailored support.

Four studies used online platforms. Online platforms were used for a range of purposes, including tracking health symptoms^36^, improving mental health^39^, and improving health education and decision making^24,41^. The duration of engagement with the online platforms ranged from a single 45 min session, to 60 days of use. These platforms incorporated features like real-time feedback, interactive modules, and progress tracking to enhance user engagement. Whether focused on symptom logging, or behavior change, the platforms were effective in achieving their objectives by improving learning outcomes and influencing positive behavioral shifts.

One study used a text messaging intervention^37^. Text4Hope employed daily *supportive* text messages across a 3-month period, designed on behavioral cognitive therapy to reduce symptoms of depression and anxiety and improve resilience and well-being during the wildfires in Canada. This study incorporated baseline and post intervention questionnaires demonstrating improvements in wellbeing, anxiety and resilience.

### Study outcomes

**Fig. 4:**
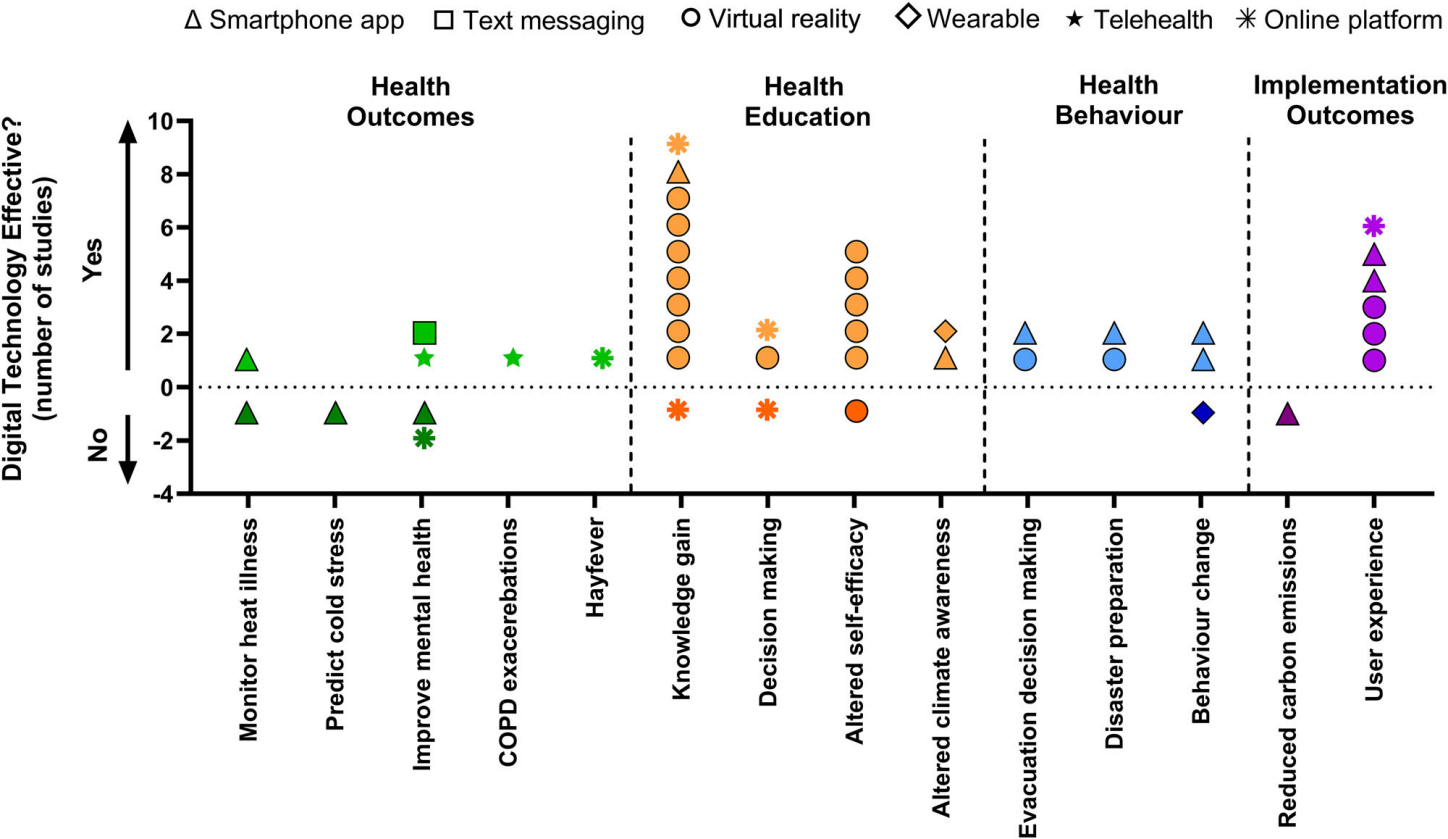
Representation of outcome measures, type of digital technology and efficacy. Health outcome measures are presented in green, health education in orange, behavioral outcome measures in blue and implementation outcome measures in purple. Dark colors indicate that digital technology was not effective at achieving that outcome measure, light colors indicate it was effective. The dashed line denotes effective interventions and dark colors below the dashed line denote ineffective or detrimental interventions. Symbol types depict different technologies employed, specifically smartphone apps (triangles), text messaging (squares), virtual reality (circles), telehealth (pentagonal stars), wearable technology (diamonds), and online platforms (octagonal stars).

Outcome measures for the included studies were classified into health outcomes, health education, behavior, and/or implementation. Figure 4 depicts the outcomes measures and effectiveness of digital technology to achieve the aim of each selected study, with some studies utilizing multiple outcome measures.

Nine studies used digital technology for health-related outcome measures. The primary aims of these studies were to: monitor heat stress (*n* = 2), predict cold stress (*n* = 1), reduce COPD exacerbations during heatwaves (*n* = 1), reduce hay fever symptoms (*n* = 1) and manage/improve climate change-related mental health challenges such as depression, anxiety, coping or quality of life (*n* = 4). Studies utilizing wearables improved awareness and data collection but had limited impact on behavior change and heat stress detection^25,32^. Smartphone apps were both ineffective at monitoring heat illness^25^ and predicting cold stress^27^. Telehealth was effective at reducing COPD exacerbations^33^ during heatwaves, and an online platform where people could receive information/monitor their allergies was effective in reducing people’s hay fever symptoms^36^.

A 3-month text-based program (Text4Hope) used during the Canadian wildfires and an internet-based cognitive behavioral therapy program showed improvements in wellbeing, depression, anxiety and post-traumatic stress disorder symptoms^37^. Conversely, a web-based platform used to reduce depression and anxiety symptoms and improve coping during hurricane Ike, showed no improvements in any mental health outcomes measures except mitigating symptoms of worry^39^. Similarly, a mobile app aimed at improving prenatal environmental awareness was beneficial at improving health behaviors, but did not improve symptoms of depression or anxiety^34^.

Eleven studies assessed the use VR (*n* = 7)^20–22,26,28,29,42^, online platforms (*n* = 2)^24,41^, wearable (*n* = 1)^25^ and a smartphone application (*n* = 1)^31^ to improve health education. In almost all studies, digital technologies were effective at improving knowledge gain and self-efficacy about specific topics of interest. Topics ranged from learning the signs of heat stress and mitigation strategies^22,25^, safe evacuation strategies during earthquakes^20,21,28,29^ to learning how to prepare for and protect oneself during wildifres^24^. Only one study demonstrated no increase in self-efficacy following a VR simulation to improve earthquake evacuation safety^29^.

Six studies used digital technology to alter behavior and/or improve decision making (Fig. 4). Some studies used digital technology to alter how people respond to and/or the time it takes to make a decision and effective evacuation strategies for disasters, such as tsunamis, floods and hurricanes^19,23,30^. For these studies, digital technology was effective in improving behavioral responses to emergency situations. Smartphone apps were also used for behavior change and were effective at changing people’s diet behaviors^38^ and environmental health behavior^34^, yet demonstrated no change in motivation or intention to change travel behaviors despite increased awareness of their surrounding air pollution^32^.

Seven studies assessed the efficacy of digital technology implementation. The main outcomes for implementation were user experience (*n* = 6) and reduction in carbon emissions (*n* = 1). In all studies, the use of digital technologies had high satisfaction for user experience. One study used education through an app to change people’s diet choices and ultimately reduce greenhouse gas emissions from food waste but showed no reductions in greenhouse gas emissions (Fig. 4, Supplementary Table 1).

### Semantic analysis of abstracts

Supplementary Fig. 1 is a visual depiction of the semantic analysis of abstracts of included studies. Clusters are indicative of the strength of association between words. Cluster 1 (purple, ten items) focuses on climate change and sustainability. Cluster 2 (dark blue, ten items) focuses on types of study and outcomes like virtual reality, knowledge and self-efficacy. Cluster 3 (green, seven items) focused on heat and wearables. The term ‘intervention’ was the most frequently used in all abstracts.

## Discussion

This systematic scoping review demonstrated that digital technologies are effective at improving health-related outcomes, education and behaviors in the context of climate change. In general, this is a young research topic and there is a clear scarcity of research on the application and implementation of digital health technologies in the context of human health and climate change. Ostensibly, digital technology would be used for health monitoring and tracking as well as information dissemination during extreme weather events, yet from the published works, most technologies are used for the purpose of health education, with little evidence of their efficacy for health monitoring. This highlights the need to develop and expand publicly available technologies to provide climate-related health advice and enhance community resilience in the face of a rapidly changing climate.

The first study included in our analysis was published in 2012, in contrast to similar reviews identifying studies from 1961 (cardiovascular disease)^10^ and 1998 (diabetes)^43^. While these fields are more established, interest in digital technologies within these areas has grown substantially, with publications on cardiovascular disease increasing from just one in 1961 to over 2000 by 2020^10^. Comparatively, our review found only 8 publications on climate change and digital health in 2023. Similarly, while large author collaborations exist in digital technology for other fields^10^, no collaboration between researchers were identified in the current review. As a result of the numerous technologies being used and the lack of collaboration among researchers, it is likely that critical lessons being learned in one technological (or climatic, e.g., heatwaves vs floods) space are not being transferred to other spaces. In our analysis 20 of the 24 studies originated from countries in the Global North. Given that it is expected that climate change will primarily affect countries from the Global South^44^, more research from these countries is needed.

The lack of co-design (only 3/24 studies) of digital technologies was a notable oversight in almost all included studies - a finding that has been previously demonstrated^10^. Co-design and patient-centered care are essential for effective implementation and to overcome problems faced when engaging with culturally and linguistically diverse populations^45^, adolescents and the elderly^13^. Studies included in this review demonstrated an equal mix of female and male participants. However, in general, there was a lack of research targeting individualized care to support populations who are disproportionately impacted by climate change such as people living in poverty, in rural/remote areas or with chronic diseases^5,46^. These are also the populations who are most impacted by the digital divide, being disadvantaged by the use, access and affordability of digital technologies. This gap highlights the urgent need for more inclusive research and interventions that prioritize the most vulnerable populations, ensuring equitable access to and benefits from digital health innovations in the face of climate change

Our initial search uncovered a variety of technologies designed to monitor health outcomes, but only nine studies were included in the final analysis. Most studies were excluded because their technology was in the early proof-of-concept phase or focusing on biometric data without providing actionable insights to protect health. When responding to climatic events, multiple interventions are often applied simultaneously, making it difficult to assess the impact of any single technology^47^. This context highlights a gap in the application of traditional communication tools, such as radio and television, which are typically used for broad public health messaging. While these tools can raise awareness, they often lack the ability to provide personalized, timely guidance needed for effective health monitoring. In contrast, a systematic review of 11 heat action plans found that successful interventions prioritized direct communication with vulnerable populations via text or phone, emphasizing the importance of tailored messaging in promoting health outcomes during climate events^48^. By integrating health monitoring technologies with direct communication strategies, such as text messaging or mobile applications that provide real-time alerts and tailored advice, we could better equip healthcare providers and vulnerable populations to respond effectively to climate-related health challenges.

Only three studies included in our analysis used wearables. This finding contrasts with a recent scoping review on the use of wearables in climate change, which included 53 papers^49^. The major discrepancy between the two reviews was the requirement in the current review for wearables to provide actionable recommendations to protect health and not simply measure biometrics. Wearables, like smartwatches, trackers and rings have surged in popularity. Problematically, most wearables remain in the design or prototype phase and/or have not been applied in a real-world settings^49^. Many wearables collect physiological data such as skin temperature and heart rate and are therefore theoretically capable of monitoring climate-related health risks like heat stroke^49^. However, there are important physiological and technical aspects that need to be considered when interpreting physiological measurements drawn from wearables. For example, core temperature estimates taken from the skin surface do not agree well with more reliable measurements of core temperature, and are often heavily influenced by ambient temperature and physical activity^50^.

Smartphones, apps, text messaging systems, and telehealth can integrate scientific and clinical data to assess an individual’s climate-related health risks^32^. Online and game-based learning has also gained attention to improve health education. Most studies included in this review focused on health education. These studies were effective at educating people on how to protect themselves during flooding^26^ events, or how safely evacuate from earthquakes^28,29^. Other studies focused on educating people in the impacts of climate change events on their health, such as heatwaves^25^ or wildfires^24^. Fewer studies looked at the influence of digital technology to alter health behaviors. Of the studies that did, digital technology seemed effective at influencing people’s intention to change their behavior when preparing for and making decisions regarding evacuation^23,30^, or altering general health behaviors (i.e., diet)^34,38^.

Importantly, this review demonstrates all these technologies remain underutilized in the context of climate change and its health impacts. Smartphones can combine environmental data with personal health monitors and offer local warnings, physiological alerts, and actionable recommendations to protect health^10^. They can also provide education on topics like medication storage and activity limitations during extreme conditions, or automatically broadcast distress signals in emergencies, such as elevated heart rates or falls. Telehealth, which has gained traction in chronic disease management, has primarily been studied to reduce healthcare-related greenhouse gas emissions, particularly by cutting down on patient travel^33^. However, less attention has been given to telehealth’s potential for improving climate change-related health outcomes^51^. One example of its potential is the Australian Red Cross Telecross REDi Program^52^, which provides outreach during extreme heat. Volunteers call housebound elderly individuals and visit them if they do not respond, offering a lifeline to those most vulnerable during heatwaves highlighting how digital health can be leveraged to protect at-risk populations during climate-related events.

Social media has the potential to help communities prepare for and manage extreme weather disasters. Algorithms now analyze posts, engagement, and location to track and predict disaster magnitude and response needs, especially in rural areas where monitoring is limited. For instance, Twitter sentiment analysis accurately predicted wildfire speed and location but struggled with size and spread^47^. Conversely, in Croatia, integrating social media and crowd-sourced data improved fire response tracking^53^. These methods aim to enhance early warning systems and disaster response efficiency through social media nowcasting; however, studies to date are still in the development phase, and the impact social media has on mitigating negative health outcomes is unknown. We did not identify any research that used social media to reduce the impacts of climate change on health. As social media consumption grows and there is an increased societal reliance on its use, there is an immense potential to reach and target a large audience with both climate awareness messaging and early warning messaging in the event of a climate disaster.

Because our search terms required studies to include health, behavior, or education outcomes, some technologies and studies related to climate events were likely excluded from this review. For instance, while public health advice is often broadcast through radio and television during such events, these methods were not included in our analysis. Similarly, early warning systems and personal-use technologies like weather stations, thermometers, and air pollution meters, which were designed but not used to monitor personal health metrics, were also not represented. Therefore, this review offers new insights into the use of digital technologies in climate space, but it does not cover all existing technologies or their applications in indirectly protecting people before and during climate events. Finally, following established guidelines for scoping reviews^54^, we did not conduct a formal quality appraisal of the included studies. As a result, some conclusions drawn from these studies may be affected by methodological limitations (e.g., an intervention reported as ineffective may have been underpowered). This should be considered when interpreting the findings of this review.

This review demonstrates that digital technologies are effective at improving climate change-related health outcomes, education and behaviors. However, the use of digital technologies to inform and protect the public from climate change-induced events is still emerging. As more technologies come into existence, the potential to educate and protect the public has never been greater. This is particularly true for those who may have limited access to care, live socially isolated lives or reside in rural/remote areas. In general, studies were primarily conducted in globally northern countries, with no collaboration between different research teams/sectors. Future studies in this area should focus on collaborative studies that are co-designed with the end-users of technology.

## Methods

### Search strategy

A literature search was performed to identify all available studies on digital technologies and climate change. An initial search string was developed based on a recently published review on the use of digital technologies and cardiovascular disease^10^, supplementing cardiovascular terms for heatwave and extreme heat terms. After an initial exploration of the search string results, the search string was updated to incorporate all climate change-derived disasters. This iterative refinement is consistent with established scoping review methodology^55,56^. Subsequently, a University of Colorado Librarian helped translate the search string for PubMed, using the appropriate meSH terms (available in the supplementary materials file). The systematic search was conducted in Web of Science and PubMed, and included articles published until March 11th, 2025. The search was conducted using a list of key search terms identified and agreed upon by the authors and organized into a Boolean search strategy. The protocol was developed using a scoping review framework^54^, and followed the Preferred Reporting Items for Systematic Reviews and Meta Analyses’ reporting guidelines for scoping reviews (Supplementary Table 2)^57^. Given the iterative nature of the scoping review process, and because preregistration is not a standard component of a scoping review, the current review was not preregistered^54^.

### Eligibility criteria

Studies were included if they 1) were published in English, regardless of publication date 2) were an original research article conducted on humans, and 3) tested the efficacy of a technology for either a) improving health outcomes, b) modifying behavior or decision making related to health or c) health education. While an increased risk of earthquakes is not directly related to climate change, studies on this topic area were still included due to recent evidence that suggests rising air and water temperatures, glacial melting and rising sea levels influence seismic activity^58,59^. Similarly in relation to tsunamis, climate change-related increases in seismic and volcanic activity, alongside landslides and rising sea levels also increase the risk of tsunamis^58,60^. Because of this, both these natural disasters were included in our search. Studies that collected metrics on the implementation of the intervention were only included if one of the former efficacy outcomes were also measured (i.e., studies that only measured implementation metrics were excluded). Any wearable, sensor or other technology that was in the design/prototype phase and had not been tested or validated on humans was excluded from the analysis. Furthermore, wearables were only included if they provided actionable health recommendations based on the biometrics measured and were not solely used for the purpose of biometric monitoring in simulated climate change environments. Studies were also excluded if they were only a survey, review, conference paper, case study, or the digital health technology being used was not explicitly linked to a climate change-related outcome. Telehealth papers were only included where they discussed health outcomes related to climate change and not just changes in carbon emissions.

To identify relevant papers, titles and abstracts were screened in duplicate by two teams consisting of either GC and MB or NM and AR using Rayyan screening software. Following title screening, GC and NM reviewed and resolved any conflicts between the teams. Data extraction was performed in duplicate with the following metrics extracted using a standardized excel template: First Author, year of publication, country of origin, study aim, methodology, presence of co-design, type of outcome (e.g. health outcomes, behavior change, health education or implementation), digital technology used, population, climate change topic, and the efficacy of the digital technology. For type of outcome, studies were categorized as health outcomes if the main purpose of the paper was to use digital technology to improve health outcomes affected by climate change. Studies were categorized as behavior change if the main purpose of the study was focused on behavior change or altered decision making that would have health implications (e.g. safely evacuating an earthquake). Studies were categorized as health education where the main purpose of the study was to educate on health outcomes related to climate change. Lastly, studies were categorized as implementation if secondary outcomes of the study focused on the improvement of digital technology itself. All data are presented as the mean and SD unless stated otherwise.

### Semantic analysis

To gain further insight into general patterns in the field of digital health technologies, we performed two additional scientometric analyses of the data. Firstly, an abstract analysis was performed to identify clusters of terms based on their frequency of occurrence within the abstract section of each study. Due to the limited sample size, the minimum term frequency was set to five. Secondly, an analysis of author collaboration, where each author is represented by a node and collaboration between authors is represented by a conjoining link, with the link thickness denoting the number of co-authored documents. These semantic analyses were conducted using VOSviewer (v1.6.20, Leiden University, Netherlands).

## Supplementary information

Supplementary Information

## Data Availability

All data generated or analyzed during this study are included in this published article and its supplementary information files, and the original data and detailed analysis methods can be shared upon reasonable request to the corresponding author. VOSviewer interactive maps are available using the following links for the author (https://app.vosviewer.com/?json=https%3A%2F%2Funisyd-my.sharepoint.com%2F%3Au%3A%2Fg%2Fpersonal%2Fgeorgia_chaseling_sydney_edu_au%2FEVbquMitQTlLhWTzjJGEh8wBVCrJFJbWAO6Pw3KS7-NGBA%3Fdownload%3D1) and abstract (https://app.vosviewer.com/?json=https%3A%2F%2Funisyd-my.sharepoint.com%2F%3Au%3A%2Fg%2Fpersonal%2Fgeorgia_chaseling_sydney_edu_au%2FEcAcTrKFt3NOvOrM0HRy3gMBa9WLMzMPbD2sH7r-2RPwOA%3Fdownload%3D1) data.
