# Supplementary material for: Digital technologies for climate-related health education, behavior and risk reduction: a systematic scoping review
Source: NPJ Digit Med. 2025 Aug 2;8:498. doi: 10.1038/s41746-025-01907-5 (PMC12318067; doi:10.1038/s41746-025-01907-5)
Supplement: Supplementary file 1 — Supplementary Information [file 41746_2025_1907_MOESM1_ESM.pdf]

Digital technologies for climate-related health education, behavior and risk reduction to reduce climate change-related health risks: A systematic scoping review – Supplementary Material

Nathan B Morris[1], Megan Barnes[1], Autumn Rybarczyk[1], Georgia K Chaseling[2,3] ✉

[1]William J. Hybl Sports Medicine and Performance Center, Department of Human Physiology and Nutrition, University of Colorado, Colorado Springs, Colorado, USA

[2]Sydney Nursing School, Faculty of Medicine and Health, The University of Sydney, Sydney, NSW, AUS

[3]Heat and Health Research Centre, School of Health Sciences, Faculty of Medicine and Health, The University of Sydney, Sydney, NSW, AUS

Address for correspondence:
Georgia Chaseling, PhD
Sydney Nursing School
University of Sydney
Susan Wakil Health Building, Western Ave,
Camperdown, NSW, 2050
Email: georgia.chaseling@sydney.edu.au

Table of contents

Search strings 3

Supplementary Table 1. Summary of included studies. 6

Supplementary Figure 1. Semantic analysis of abstracts of accepted manuscripts 14

Supplementary Table 2. PRISMA-ScR Checklist 15

Search strings

Web of Science – 18,382 results

TI=((Heat OR heatwave* OR “hot weather” OR “heat health” OR hyperthermia OR “climate change” OR flood* OR wildfire* OR “natural disaster*” OR cyclone* OR “bush fire*” OR bushfire* OR “grass fire*” OR grassfire* OR landslide* OR mudslide* OR “wind storm*” OR windstorm* OR tornado* OR “hail storm*” OR hailstorm* OR earthquake* OR rainstorm* OR typhoon* OR hurricane* OR “forest fire*” OR “snow melt*” OR snowmelt* OR “air pollution” OR smog OR “cold snap*” OR “cold stress” OR tsunami* OR “tidal wave*” OR blizzard* OR “extreme weather” OR “weather event*”) AND (“health tech*” OR “healthcare tech*” OR “electronic healthcare” OR “digital healthcare” OR cybermedicine OR cybertherapy OR telehealth OR telemedic* OR ehealth OR “electronic health” OR “digital health” OR emedicine OR “digital medicine” OR mhealth OR “mobile health” OR “electronic prescribing” OR “E-prescription” or eprescription* OR evisit OR “E-visit” OR “video-assisted” OR “video health” OR “health information technology” OR “health information technologies” OR telecardiology OR telemonitor* OR telerehabilitation OR “health monitoring system” OR “healthcare monitoring system” OR “health monitoring device” OR "mobile app*" OR smartphone* OR iphone OR ipad OR iOS OR “mobile phone*” OR “cell phone*” OR “android phone*” OR “text message” OR “text messaging” OR “text-messaging” OR “short message service” OR SMS OR chatbot* OR “smart sensor” OR “body sensor*” OR “wireless sensor*” OR “health tracker*” OR “fitness tracker*” OR “fitness app*” OR “sleep tracking device” OR “fitness watch” OR “apple watch” OR fitbit OR garmin OR “activity tracker*” OR wearable OR smartwatch OR “personal monitoring device” OR “web-based” OR “web based” OR “online community” OR “online platform*” OR webinar* OR avatar* OR Twitter OR “social media” OR “Youtube” OR “Instagram” OR “facebook” OR tiktok OR “videogame*” OR “video game*” OR “computer game*” OR wii OR gamification OR “immersive tech*” OR “virtual realit*” OR “augmented realit*” OR “mixed realit*”)) OR

AB=((Heat OR heatwave* OR “hot weather” OR “heat health” OR hyperthermia OR “climate change” OR flood* OR wildfire* OR “natural disaster*” OR cyclone* OR “bush fire*” OR bushfire* OR “grass fire*” OR grassfire* OR landslide* OR mudslide* OR “wind storm*” OR windstorm* OR tornado* OR “hail storm*” OR hailstorm* OR earthquake* OR rainstorm* OR typhoon* OR hurricane* OR “forest fire*” OR “snow melt*” OR snowmelt* OR “air pollution” OR smog OR “cold snap*” OR “cold stress” OR tsunami* OR “tidal wave*” OR blizzard* OR “extreme weather” OR “weather event*”) AND (“health tech*” OR “healthcare tech*” OR “electronic healthcare” OR “digital healthcare” OR cybermedicine OR cybertherapy OR telehealth OR telemedic* OR ehealth OR “electronic health” OR “digital health” OR emedicine OR “digital medicine” OR mhealth OR “mobile health” OR “electronic prescribing” OR “E-prescription” or eprescription* OR evisit OR “E-visit” OR “video-assisted” OR “video health” OR “health information technology” OR “health information technologies” OR telecardiology OR telemonitor* OR telerehabilitation OR “health monitoring system” OR “healthcare monitoring system” OR “health monitoring device” OR "mobile app*" OR smartphone* OR iphone OR ipad OR iOS OR “mobile phone*” OR “cell phone*” OR “android phone*” OR “text message” OR “text messaging” OR “text-messaging” OR “short message service” OR SMS OR chatbot* OR “smart sensor” OR “body sensor*” OR “wireless sensor*” OR “health tracker*” OR “fitness tracker*” OR “fitness app*” OR “sleep

tracking device” OR “fitness watch” OR “apple watch” OR fitbit OR garmin OR “activity tracker*” OR wearable OR smartwatch OR “personal monitoring device” OR “web-based” OR “web based” OR “online community” OR “online platform*” OR webinar* OR avatar* OR Twitter OR “social media” OR “Youtube” OR “Instagram” OR “facebook” OR tiktok OR “videogame*” OR “video game*” OR “computer game*” OR wii OR gamification OR “immersive tech*” OR “virtual realit*” OR “augmented realit*” OR “mixed realit*”)) OR

AK=((Heat OR heatwave* OR “hot weather” OR “heat health” OR hyperthermia OR “climate change” OR flood* OR wildfire* OR “natural disaster*” OR cyclone* OR “bush fire*” OR bushfire* OR “grass fire*” OR grassfire* OR landslide* OR mudslide* OR “wind storm*” OR windstorm* OR tornado* OR “hail storm*” OR hailstorm* OR earthquake* OR rainstorm* OR typhoon* OR hurricane* OR “forest fire*” OR “snow melt*” OR snowmelt* OR “air pollution” OR smog OR “cold snap*” OR “cold stress” OR tsunami* OR “tidal wave*” OR blizzard* OR “extreme weather” OR “weather event*”) AND (“health tech*” OR “healthcare tech*” OR “electronic healthcare” OR “digital healthcare” OR cybermedicine OR cybertherapy OR telehealth OR telemedic* OR ehealth OR “electronic health” OR “digital health” OR emedicine OR “digital medicine” OR mhealth OR “mobile health” OR “electronic prescribing” OR “E-prescription” or eprescription* OR evisit OR “E-visit” OR “video-assisted” OR “video health” OR “health information technology” OR “health information technologies” OR telecardiology OR telemonitor* OR telerehabilitation OR “health monitoring system” OR “healthcare monitoring system” OR “health monitoring device” OR "mobile app*" OR smartphone* OR iphone OR ipad OR iOS OR “mobile phone*” OR “cell phone*” OR “android phone*” OR “text message” OR “text messaging” OR “text-messaging” OR “short message service” OR SMS OR chatbot* OR “smart sensor” OR “body sensor*” OR “wireless sensor*” OR “health tracker*” OR “fitness tracker*” OR “fitness app*” OR “sleep tracking device” OR “fitness watch” OR “apple watch” OR fitbit OR garmin OR “activity tracker*” OR wearable OR smartwatch OR “personal monitoring device” OR “web-based” OR “web based” OR “online community” OR “online platform*” OR webinar* OR avatar* OR Twitter OR “social media” OR “Youtube” OR “Instagram” OR “facebook” OR tiktok OR “videogame*” OR “video game*” OR “computer game*” OR wii OR gamification OR “immersive tech*” OR “virtual realit*” OR “augmented realit*” OR “mixed realit*”))

PubMed – 4,903 results

((("Hot Temperature"[Mesh] OR "Extreme Heat"[Mesh] OR "Heat Stress Disorders"[Mesh] OR "Heat-Shock Response"[Mesh] OR "Climate Change"[Mesh] OR Floods[Mesh] OR Wildfires[Mesh] OR “Natural Disasters” [Mesh] OR “Cyclonic Storms” [Mesh] OR Landslides[Mesh] OR Tornadoes[Mesh] OR Earthquakes[Mesh] OR “Air Pollution” [Mesh] OR Smog[Mesh] OR “Cold Temperature” [Mesh] OR “Extreme Cold”[Mesh] OR “Cold-Shock Response” [Mesh] OR Tsunamis[Mesh] OR “Tidal Waves” [Mesh] OR Snow[Mesh] OR “Extreme Weather”[Mesh]) OR (Heat[Title/Abstract] OR heatwave*[Title/Abstract] OR "hot weather"[Title/Abstract] OR "heat health"[Title/Abstract] OR hyperthermia[Title/Abstract] OR "climate change"[Title/Abstract] OR flood*[Title/Abstract] OR wildfire*[Title/Abstract] OR “natural disaster*”[Title/Abstract] OR cyclone*[Title/Abstract] OR “bush fire*”[Title/Abstract] OR bushfire*[Title/Abstract] OR “grass fire*”[Title/Abstract] OR grassfire*[Title/Abstract] OR landslide*[Title/Abstract] OR

mudslide*[Title/Abstract] OR avalanche*[Title/Abstract] OR “wind storm*”[Title/Abstract] OR windstorm*[Title/Abstract] OR tornado*[Title/Abstract] OR “hail storm*”[Title/Abstract] OR hailstorm*[Title/Abstract] OR earthquake*[Title/Abstract] OR rainstorm*[Title/Abstract] OR typhoon*[Title/Abstract] OR hurricane*[Title/Abstract] OR “forest fire*”[Title/Abstract] OR “snow melt*”[Title/Abstract] OR snowmelt*[Title/Abstract] OR “air pollution” [Title/Abstract] OR smog[Title/Abstract] OR “cold snap*”[Title/Abstract] OR “cold stress” [Title/Abstract] OR tsunami*[Title/Abstract] OR “tidal wave*”[Title/Abstract] OR blizzard*[Title/Abstract] OR “extreme weather”[Title/Abstract] OR “weather event”[Title/Abstract])) AND (("Telemedicine"[Mesh] OR "Cell Phone"[Mesh] OR "Digital Health"[Mesh] OR "Virtual Reality"[Mesh] OR "Video Games"[Mesh] OR "Internet"[Mesh]) OR ("health tech*"[Title/Abstract] OR "healthcare tech*"[Title/Abstract] OR "electronic healthcare"[Title/Abstract] OR "digital healthcare"[Title/Abstract] OR cybermedicine[Title/Abstract] OR cybertherapy[Title/Abstract] OR telehealth[Title/Abstract] OR telemedic*[Title/Abstract] OR ehealth[Title/Abstract] OR "electronic health"[Title/Abstract] OR "digital health"[Title/Abstract] OR emedicine[Title/Abstract] OR "digital medicine"[Title/Abstract] OR mhealth[Title/Abstract] OR "mobile health"[Title/Abstract] OR "electronic prescribing"[Title/Abstract] OR "E-prescription"[Title/Abstract] OR eprescription*[Title/Abstract] OR evisit[Title/Abstract] OR "E-visit"[Title/Abstract] OR "video-assisted"[Title/Abstract] OR "video health"[Title/Abstract] OR "health information technology"[Title/Abstract] OR "health information technologies"[Title/Abstract] OR telecardiology[Title/Abstract] OR telemonitor*[Title/Abstract] OR telerehabilitation[Title/Abstract] OR "health monitoring system"[Title/Abstract] OR "healthcare monitoring system"[Title/Abstract] OR "health monitoring device"[Title/Abstract] OR "mobile app*"[Title/Abstract] OR smartphone*[Title/Abstract] OR iphone[Title/Abstract] OR ipad[Title/Abstract] OR iOS[Title/Abstract] OR "mobile phone*"[Title/Abstract] OR "cell phone*"[Title/Abstract] OR "android phone*"[Title/Abstract] OR "text message"[Title/Abstract] OR "text messaging"[Title/Abstract] OR "text-messaging"[Title/Abstract] OR "short message service"[Title/Abstract] OR SMS[Title/Abstract] OR chatbot*[Title/Abstract] OR "smart sensor"[Title/Abstract] OR "body sensor*"[Title/Abstract] OR "wireless sensor*"[Title/Abstract] OR "health tracker*"[Title/Abstract] OR "fitness tracker*"[Title/Abstract] OR "fitness app*"[Title/Abstract] OR "sleep tracking device"[Title/Abstract] OR "fitness watch"[Title/Abstract] OR "apple watch"[Title/Abstract] OR fitbit[Title/Abstract] OR garmin[Title/Abstract] OR "activity tracker*"[Title/Abstract] OR wearable[Title/Abstract] OR smartwatch[Title/Abstract] OR "personal monitoring device"[Title/Abstract] OR "web-based"[Title/Abstract] OR "web based"[Title/Abstract] OR "online community"[Title/Abstract] OR "online platform*"[Title/Abstract] OR webinar*[Title/Abstract] OR avatar*[Title/Abstract] OR Twitter[Title/Abstract] OR "social media"[Title/Abstract] OR "Youtube"[Title/Abstract] OR "Instagram"[Title/Abstract] OR "facebook"[Title/Abstract] OR tiktok[Title/Abstract] OR "videogame*"[Title/Abstract] OR "video game*"[Title/Abstract] OR "computer game*"[Title/Abstract] OR wii[Title/Abstract] OR gamification[Title/Abstract] OR "immersive tech*"[Title/Abstract] OR "virtual realit*"[Title/Abstract] OR "augmented realit*"[Title/Abstract] OR "mixed realit*"[Title/Abstract])))

Supplementary Table 1. Summary of included studies.

Reference	Climate Change Topic	Digital Technology	Study Aim	Outcome Measures	Population	Methodology	Main Findings
Aydoğan *et al* 2022 Turkey	Climate change	Smartphone app	Develop and test an Android mobile app as an effective smart learning environment for climate change health impacts.	**Health education:** Climate change awareness and impact on health<br><br>**Implementation:** User experience	**N:** 44 (13M/31F)<br>**Age (y):** NA<br>**Demographic:** Students from department of Education and Biology, Turkey University	**Design:** A 31-question test on climate change awareness was given before and after participants used the app. A usability questionnaire was used to evaluate the effectiveness of app.<br>**Duration:** Not provided<br>**Control:** No<br>**Co-Designed:** No	The app improved climate change awareness and impact on health. Students' learning experience was well received in terms of adopting and using it for educational purposes.
Chavez Santos *et al* 2022 United States	Heatwaves	Smartphone app/ wearable	Evaluate the efficacy of farmworker participatory heat education and a supervisor decision-support mobile application (HEAT intervention) on physiological heat stress.	**Health outcome:** Monitor heat stress	**N:** INT = 37 (27M/10F) CON = 38 (21M/17F)<br>**Age (y):** 18+<br>**Demographic:** Fruit and vineyard workers in Washington, USA	**Design:** Participants took a baseline survey on heat risks and filled out weekly symptom surveys. Supervisors used the HEAT app to educate workers. Tympanic temperature and heart rate were recorded at each shift's start.<br>**Duration:** May - August 2019<br>**Control:** Workers who did not receive either the HEAT education or access to the HEAT app<br>**Co-Designed:** Yes	Heat stress (tympanic temperature and heart rate) was not different between groups. Self-reported heat symptoms did not overlap with physiological measures of heat stress.
Eggling *et al* 2023 Netherlands	Extreme cold	Smartphone app	Assess the thermal stress prediction of ClimApp in cold environments based on thermal perception.	**Health outcome:** Predict cold stress	**N:** 55 (28M/27F)<br>**Age (y):** 32 ± 11<br>**Demographic:** Healthy adults	**Design:** Participants exposed to an outdoor environment of >10°C recording their activity in ClimApp. Cold stress was calculated and compared to the participants perceived thermal sensation.<br>**Duration:** 60 minutes<br>**Control:** No<br>**Co-Designed:** No	App could predict thermal sensation, but not accurately predict cold thermal stress.
Han *et al* 2023 Japan	Tsunami	Smartphone app	Assess the efficacy of an app on decision making and ability to	**Behavior:** Decision making during a tsunami evacuation	**N:** 43 (20M/23F)<br>**Age (y):** 15-27	**Design:** Two groups of participants were required to seek refuge during a simulated tsunami; one group was	The number of participants who reached refuge was similar between

			reach safety during a Tsunami evacuation		**Demographic:** Japanese residents residing away from area of evacuation	provided with a smartphone app for aid.<br>**Duration:** Time taken to find refuge<br>**Control:** No smartphone app<br>**Co-Designed:** No	groups. Time taken and distance travelled to reach refuge was shorter for people using the smartphone app.
Jo *et al*<br>2025<br>South Korea	Climate change	Smartphone app	Assess DECO-MOM app on health behaviors, mental health and quality of life	**Health outcome:** Improve mental health<br><br>**Behavior:** Environmental health behavior<br><br>**Implementation:** User experience	**N:** 65 (0M/65F)<br>**Age (y):** ~33<br>**Demographic:** Pregnant women with a gestational age of 24-48 weeks	**Design:** Non-randomized controlled pilot design with pre- and post-test assessment. Women underwent 4 weeks of education through a smartphone app divided into weekly themes that looked at environmental risks and health behaviors. **Duration:** 4-weeks<br>**Control:** Treatment as usual – in person classes<br>**Co-Designed:** No	Compared to the control group, the app improved environmental health behaviors and improved quality of life. Despite user satisfaction with the app, no improvements in anxiety, depression or subjective health status were seen
Kim *et al*<br>2022<br>United States	Heatwaves	Smartphone app/ wearable	Assessment of a biopatch to measure skin temperature, heart rate and ECG for continuous heat stress monitoring through an app.	**Health outcome:** Monitor heat stress<br><br>**Implementation:** User experience	**N:** NA<br>**Age (y):** NA<br>**Demographic:** Farmworkers from two communities in northeast central Florida	**Design:** Evaluation was done on participants who completed a set of simulated tasks, for 2 min with 1-min breaks between each task. Validation was done in farm workers across various operations.<br>**Duration:** Three trials, 15 min each, at 6:30AM, 11:00AM, and 3:00 PM<br>**Control:** No<br>**Co-Designed:** No	Heart rate showed accuracy against a polar watch. Skin temperature was not a good surrogate for core temperature<br>Efficacy of service delivery was useful for health monitoring in the field.
Pitt *et al*<br>2023<br>Sweden	Climate change	Smartphone app	Evaluate app-based dietary intervention to change dietary habits and associated changes in diet-related greenhouse gas emissions in persons with Type 2 diabetes.	**Behavioral:** Change diet behavior<br><br>**Implementation:** Reduced carbon emissions	**N:** INT=46 (18F/28M)<br>CON=47 (19F/28M)<br>**Age (y):**<br>INT = 64 ± 10<br>CON = 63 ± 11<br>**Demographic:** older adults with Type 2 diabetes	**Design:** Participants were provided education on healthy eating behaviors and were assessed pre and post intervention compared to a control group.<br>**Duration:** 12 weeks<br>**Control:** Control group received standard care without diet counselling.<br>**Co-Designed:** No	There were no changes in diet compared to baseline and therefore corresponding greenhouse gas emissions were not reduced.

Aksa *et al*<br>2025<br>United States	Flooding	Virtual reality	Assess the use of VR to improve flood evacuation knowledge and self-efficacy regarding flood disasters	**Health education:** Improve knowledge and self-efficacy about what to do during a flood evacuation.	**N:** 45 (17M/28F)<br>**Age (y):** NA<br>**Demographic:** Geography major university students residing in Indonesia	**Design:** Participants underwent VR training for safe flood evacuation (not described) and answered a questionnaire relating to self-efficacy and orally answered questions relating to knowledge of evacuation pre and post training<br>**Duration:** Not provided<br>**Control:** No<br>**Co-Designed:** No	VR improved self-efficacy and knowledge of safe flood evacuation compared to pretests.
Alzarrad *et al*<br>2023<br>United States	Heatwaves	Virtual reality	Assess the use of VR to educate construction workers on how to mitigate heat stress	**Health education:** Reduce heat stress	**N:** 82<br>**Age (y):** NA<br>**Demographic:** Construction workers	**Design:** Participants received training on the signs of heat stress and mitigation strategies either through PowerPoint or VR. Participants took a pre and post test and were marked on the number of wrong answers<br>**Duration:** Not provided<br>**Control:** Training through PowerPoint<br>**Co-Designed:** No	Compared to training through PowerPoint, participants who received training through VR reduced the number of wrong answers they received post training.
Ahmadi *et al*<br>2024<br>Iran	Earthquake	Virtual reality/ gaming	To assess a VR game on earthquake evacuation safety knowledge and self-efficacy	**Health education:** Knowledge and self-efficacy about what to do during an earthquake evacuation.	**N:** 145 (85M/60F)<br>**Age (y):** 18-33<br>**Demographic:** undergraduate and postgraduate university students	**Design:** A VR game simulated an earthquake in a shopping mall and users had to make decisions about what to do based on receiving 1) immediate feedback, 2) post-game feedback or 3) spiral feedback method. Safety knowledge and self-efficacy were assessed pre and post, game play.<br>**Duration:** Not provided<br>**Control:** No feedback<br>**Co-Designed:** No	Compared to post game feedback only, players improved their scores of safety knowledge and self-efficacy when provided immediate or spiral feedback.
Bernhardt *et al*<br>2019<br>United States	Hurricanes	Virtual reality	Assess how a VR simulation can improve the response to, and safe seeking behaviors to hurricane warnings	**Behavior:** Intention to change behavior to prepare for hurricanes	**N:** 124<br>**Age (y):** <25<br>**Demographic:** University students	**Design:** Participants were given either traditional media products (static text and map) or asked to engage with a VR hurricane simulation and traditional media products. Questionnaires on the	Participants viewing both the VR and traditional products are significantly more likely to act in preparation for a

						participants' intentions to change behavior in response to hurricane warnings were assessed pre and post.<br>**Duration:** Not provided<br>**Control:** Static text map traditionally provided by media<br>**Co-Designed:** No	hurricane compared to those being exposed to just the traditional products.
D'Amico *et al*<br>2023<br>Italy	Flooding	Virtual reality/ gaming	Assess the effect of VR training to improve safety during a flood event	**Health education:** Improve knowledge and self-efficacy about what to do during a flood evacuation.<br><br>**Implementation:** User experience	**N:** 55 (30M/25F)<br>**Age (y):** 18-68<br>**Demographic:** Not provided	**Design: Q**uestionnaires relating to knowledge gain, self-efficacy, and use experience were provided pre and post a VR game on flood safety.<br>**Duration:** Not provided<br>**Control:** No<br>**Co-Designed:** No	Knowledge gain and self-efficacy improved. High user satisfaction with the game
Feng *et al*<br>2020<br>New Zealand	Earthquake	Virtual reality/ gaming	Evaluate the efficacy of VR to train hospital staff and visitors how to respond to an earthquake emergency	**Health education:** Knowledge and self-efficacy about what to do during an earthquake evacuation.<br><br>**Implementation:** User experience	**N:** 91 (43M/50F)<br>**Age (y):** 20-87<br>**Demographic:** Staff and visitors of Auckland City Hospital	**Design:** Participants verbally answered pre and post questions regarding safe evacuation of an earthquake, and answered a post questionnaire about use experience following 20 minutes of playing a VR game where players were required to evacuate an earthquake<br>**Duration:** 20 minutes<br>**Control:** No<br>**Co-Designed:** No	Knowledge gain and self-efficacy improved for staff and visitors. In general, the game was reported as easy to use and engaging.
Feng *et al*<br>2020<br>New Zealand	Earthquake	Virtual reality/ gaming	Evaluate the efficacy of VR to train school students how to respond to an earthquake emergency	**Health education:** Knowledge and self-efficacy about what to do during an earthquake evacuation.	**N:** 125 (79M/46F)<br>**Age (y):** 11-15<br>**Demographic:** secondary school students	**Design:** Participants were either provided with a leaflet with information on safe earthquake evacuation or played a VR game that allowed them to make decisions on safe earthquake evacuation. Students were giving pre-and-post questionnaires to assess their knowledge gain and self-efficacy<br>**Duration:** NA	Knowledge gain improved after using the VR game compared to the leaflet, yet no differences in self-efficacy were seen,

					**Control:** Information leaflet<br>**Co-Designed:** No	
Fuijimi *et al*<br>2020<br>Japan	Flooding	Virtual reality	To assess the efficacy of virtual reality on people's ability to make decisions about flood evacuation	**Behavior:** Decision making during flood evacuation.	**N:** 103 (76M/27F)<br>**Age (y):** 18-24<br>**Demographic:** Japanese students in the Department of Civil and Environmental Engineering and Architecture at Kumamoto University	**Design:** Participants engaged with a VR flash flooding simulation and were assessed on their decision to evacuation time and successful evacuation. There were five VR scenarios differentiated by verbal or visual feedback<br>**Duration:** 60 seconds<br>**Control:** No feedback<br>**Co-Designed:** No	Combined visual and verbal cues promoted to earliest and safest evacuation time. Compared to no cues, all feedback interventions were effective in promoting early evacuation decisions.
Galeote *et al*<br>2023<br>Finland	Climate change	Virtual reality/ gaming	To determine the use of VR on climate change behavior.	**Health education:** Decision making, self-efficacy, attitudes<br><br>**Implementation:** User experience	**N:** CON=35 (14M/20F/1NB)<br>VR=35 (13M/21F/1NB)<br>PC=35 (14M/20F/1NB)<br>**Age (y):** ~30<br>**Demographic:** Finnish citizens	**Design:** Participants were randomized into a VR or 3D game to go through mini games about the impact of climate change on health, the causes of climate change, and the physical manifestations of climate change. Pre and post questionnaires measured the level of interest and enjoyment in experience, attitudes and self-efficacy.<br>**Duration:** One 30–60-minute session<br>**Control:** Text-based information<br>**Co-Designed:** No	Gamification improved attitudes and self-efficacy towards climate change, however there was no difference whether that change was brought on via VR or a 3D game. Non-significant trend towards a change in decision making. VR led to higher enjoyment compared to the 3D game.
Caroca *et al*<br>2019<br>Chile	Wildfire	Online platform/ gaming	Assess the effect of an online 3D game on student's response to wildfires	**Health education:** Decision making, knowledge gain.	**N:** 10<br>**Age (y):** NA<br>**Demographic:** Undergraduate students	**Design:** Group 1 (4 students) received a pre-simulation briefing on disaster management and completed a pregame questionnaire, while Group 2 (6 students) did not. All participants completed a 45-minute simulation, followed by a debrief and postgame questionnaire.<br>**Duration:** 45 min<br>**Control:** No<br>**Co-Designed:** No	Knowledge gain was better for group 1 students who received traditional teaching and video games, however no difference in decision making was noted between groups.

Medek *et al*<br>2012<br>Australia	Pollen	Online platform	Develop an internet-based hay fever diary linked to pollen load and weather variables for hay fever sufferers and determine the relationships between temperature, rainfall, pollen count and rhino conjunctivitis symptoms.	**Health outcome:** Tracking rhino conjunctivitis symptoms	**N:** 42 (22M/20F)<br>**Age (y):** 25 – 44 y<br>**Demographic:** People with allergies	**Design:** Hay fever sufferers completed a daily online allergy symptom diary while receiving daily information about pollen and weather monitoring to inform them about increases in pollen and potential worsening of allergy symptoms. Pollen counts were then correlated with allergy symptoms.<br>**Duration:** 60 days<br>**Control:** NA<br>**Co-Designed:** No	There was a link between pollen and climate variables to symptoms of rhino conjunctivitis.
Piangiamore *et al*<br>2022<br>Italy	Climate change	Online platform/ gaming	Understand how a game can educate future citizens to adopt responsible and safe behaviors in coping with natural disasters through nudging techniques to encourage positive behavior.	**Health education:** Decision making, knowledge gain.<br><br>**Implementation:** User experience	**N:** ~17,000<br>**Age (y):** NA<br>**Demographic:** Primary school students across Italy	**Design:** The game Salvina's Adventures was implemented in schools across Italy to educate children on the causes and effects of climate change. Students and teachers completed post-test surveys regarding the enjoyment and efficacy of the game and climate change knowledge gained.<br>**Duration:** Not provided<br>**Control:** NA<br>**Co-Designed:** No	The game improved decision-making skills and knowledge gain about climate change and was engaging for students.
Steinmetz *et al*<br>2012<br>United States	Hurricanes	Online platform	Test the efficacy of the My Disaster Recovery (MDR) website to decrease negative effects and increase coping self-efficacy of hurricane Ike survivors	**Health outcome:** Improve mental health outcomes	**N:** 56 (8M/48F)<br>**Age (y):** 40-45<br>**Demographic:** Survivors of Hurricane Ike	**Design:** Participants were separated into 2 groups, one receiving the usual standard of care (information only) and the other receiving 1 month of online mental health care. Participants filled out pre and post questionnaires relating to worry, depression, perceived stress, PTSD and coping<br>**Duration:** 1 month<br>**Control:** Standard care<br>**Co-Designed:** No	Participants in the intervention group improved their sense of worry. There was no difference between scores of depression, PTSD, coping or perceived stress between groups.
Becker *et al*<br>2023<br>Germany	Air pollution	Wearable	To determine if cyclists receive information about	**Health education:** Pollution awareness	**N:** 109 (48M/61F)<br>**Age (y):** 36.33±9.68	**Design:** Participants completed a survey to assess awareness of environmental pollution as a health	Wearing the sensors and receiving feedback about exposure levels

			being exposed to particulate matter, heat, or noise on their commute would result in them changing their cycling routes.	**Behavioral:** Change commute with increased knowledge of surrounding air pollution	**Demographic:** Cyclists in Germany	hazard. They then wore sensors while cycling while commuting to provide feedback about the environmental conditions. Participants received written feedback and information on the impact of air pollution and heat on health. They completed a post-test to reassess knowledge about health hazards, behavior changes, and willingness to participate in pro-climate action.<br>**Duration:** 3 days<br>**Control:** No<br>**Co-Designed:** No	significantly increased participants' perception of particulate matter as a health threat. There were no direct effects of the intervention on intentions to choose less polluted routes.
Lindhe *et al* 2023 Sweden	Climate change	Telehealth	To assess the efficacy of an internet delivered cognitive behavioral therapy program to reduce climate change related anxiety.	**Health outcome:** Improve mental health outcomes	**N:** INT=30 (4M/25F/1PNS) CON=30 (9M/21F)<br>**Age (y):** 41 (23-73)<br>**Demographic:** Swedish citizens without a severe mental illness or ongoing psychological treatment who have developed anxiety over climate change	**Design:** Participants were randomized into either a therapist-supported internet cognitive behavioral therapy or a control condition without therapy. Therapy was delivered via online modules with feedback from the therapist. Measures of depressive symptoms, stress, and quality of life anxiety, insomnia, climate change–related distress, pro-environmental behavior was assessed.<br>**Duration:** 8 weeks<br>**Control:** No therapy<br>**Co-Designed:** No	Improvements in depression, stress, climate change anxiety and quality of life with internet-based therapy compared to control. No other measures were different.
Jehn *et al* 2013 Germany	Heatwaves	Telehealth	To assess the efficacy of a tele-monitoring system on improving clinical and functional status hot days for people with COPD.	**Health outcome:** Reduce COPD exacerbations during heatwaves	**N:** INT=32 (26M/6F), CON=30 (22M/8F)<br>**Age (y):** 64 – 69<br>**Demographic:** Stage 2-5 COPD patients who had had at least 1 exacerbation in the past year	**Design:** COPD patients were randomized into a tele-monitoring or control Group. Tele-monitoring included daily clinical status and lung function tests, and weekly 6-minute walk test all completed via telemedicine.<br>**Duration:** 9 months with daily and weekly follow-ups.	Heat stress affects clinical and functional status in COPD. Tele-monitoring reduces exacerbation frequency and health care utilization during heat stress and other periods of the year.

						**Control:** Control patients received standard care with no tele-monitoring.<br>**Co-Designed:** No	
Obuobi-Donkor *et al* 2024 Canada	Wildfires	Text messaging	Evaluate the efficacy of a text message program to support psychological wellbeing during the Canadian wildfires	**Health outcome:** Improve mental health outcomes	**N:** 150 (22M/127F/1PNS)<br>**Age (y):** 16-65<br>**Demographic:** People residing in Alberta or Nova Scotia	**Design:** Users received daily supportive text messages during the wildfires in Canada based on cognitive behavioral therapy. Participants filled in pre and post questionnaires regarding anxiety, depression, well-being and PTSD<br>**Duration:** 3-months<br>**Control:** No<br>**Co-designed:** No	Text messaging improves anxiety, wellbeing, resilience and PSTD symptoms after the 3-month intervention.

COPD: Chronic obstructive pulmonary disease; NA: Not applicable; M: male; F: female; PNS: Preferred not to say; NB: Non-binary; y: years old; PTSD: Post traumatic stress disorder; VR: virtual reality.

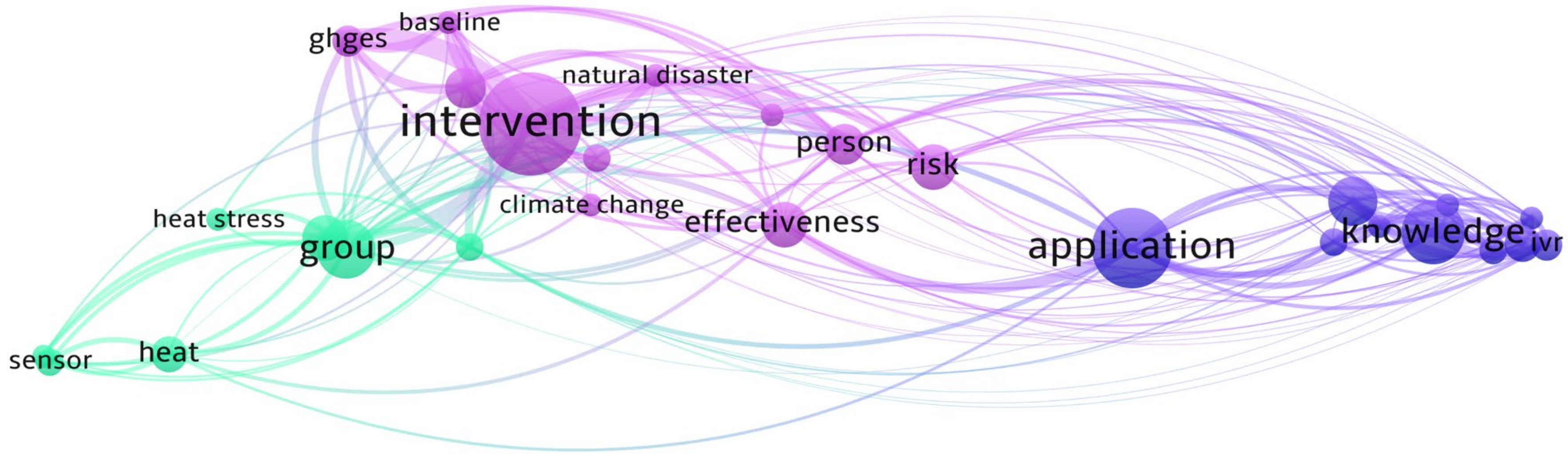

Supplementary Figure 1. Semantic analysis of abstracts of accepted manuscripts. Clusters represent the frequency of occurrence within abstract section of each paper and the thickness of lines represent the strength of association between words. IVR: Immersive virtual reality, ghges: greenhouse gas emissions.

Supplementary Table 2. PRISMA-ScR Checklist. Preferred Reporting Items for Systematic reviews and Meta-Analyses extension for Scoping Reviews (PRISMA-ScR) Checklist

SECTION	ITEM	PRISMA-ScR CHECKLIST ITEM	REPORTED ON PAGE #
**TITLE**			
Title	1	Identify the report as a scoping review.	1
**ABSTRACT**			
Structured summary	2	Provide a structured summary that includes (as applicable): background, objectives, eligibility criteria, sources of evidence, charting methods, results, and conclusions that relate to the review questions and objectives.	2
**INTRODUCTION**			
Rationale	3	Describe the rationale for the review in the context of what is already known. Explain why the review questions/objectives lend themselves to a scoping review approach.	3-5
Objectives	4	Provide an explicit statement of the questions and objectives being addressed with reference to their key elements (e.g., population or participants, concepts, and context) or other relevant key elements used to conceptualize the review questions and/or objectives.	3-5
**METHODS**			
Protocol and registration	5	Indicate whether a review protocol exists; state if and where it can be accessed (e.g., a Web address); and if available, provide registration information, including the registration number.	18
Eligibility criteria	6	Specify characteristics of the sources of evidence used as eligibility criteria (e.g., years considered, language, and publication status), and provide a rationale.	18
Information sources*	7	Describe all information sources in the search (e.g., databases with dates of coverage and contact with authors to identify additional sources), as well as the date the most recent search was executed.	18
Search	8	Present the full electronic search strategy for at least 1 database, including any limits used, such that it could be repeated.	Supplementary material
Selection of sources of evidence†	9	State the process for selecting sources of evidence (i.e., screening and eligibility) included in the scoping review.	17-19
Data charting process‡	10	Describe the methods of charting data from the included sources of evidence (e.g., calibrated forms or forms that have been tested by the team before their use, and whether data charting was done independently or in duplicate) and any processes for obtaining and confirming data from investigators.	17-19
Data items	11	List and define all variables for which data were sought and any assumptions and simplifications made.	17-19
Critical appraisal of individual sources of evidence§	12	If done, provide a rationale for conducting a critical appraisal of included sources of evidence; describe the methods used and how this information was used in any data synthesis (if appropriate).	NA
Synthesis of results	13	Describe the methods of handling and summarizing the data that were charted.	19
**RESULTS**			

SECTION	ITEM	PRISMA-ScR CHECKLIST ITEM	REPORTED ON PAGE #
Selection of sources of evidence	14	Give numbers of sources of evidence screened, assessed for eligibility, and included in the review, with reasons for exclusions at each stage, ideally using a flow diagram.	Figure 1
Characteristics of sources of evidence	15	For each source of evidence, present characteristics for which data were charted and provide the citations.	5
Critical appraisal within sources of evidence	16	If done, present data on critical appraisal of included sources of evidence (see item 12).	NA
Results of individual sources of evidence	17	For each included source of evidence, present the relevant data that were charted that relate to the review questions and objectives.	p5-14 & Supplementary material
Synthesis of results	18	Summarize and/or present the charting results as they relate to the review questions and objectives.	5-11
**DISCUSSION**			
Summary of evidence	19	Summarize the main results (including an overview of concepts, themes, and types of evidence available), link to the review questions and objectives, and consider the relevance to key groups.	12-17
Limitations	20	Discuss the limitations of the scoping review process.	17
Conclusions	21	Provide a general interpretation of the results with respect to the review questions and objectives, as well as potential implications and/or next steps.	17
**FUNDING**			
Funding	22	Describe sources of funding for the included sources of evidence, as well as sources of funding for the scoping review. Describe the role of the funders of the scoping review.	19

JBI = Joanna Briggs Institute; PRISMA-ScR = Preferred Reporting Items for Systematic reviews and Meta-Analyses extension for Scoping Reviews.
